# NOTCH Signaling Is Activated through Mechanical Strain in Human Bone Marrow-Derived Mesenchymal Stromal Cells

**DOI:** 10.1155/2019/5150634

**Published:** 2019-02-26

**Authors:** Fani Ziouti, Regina Ebert, Maximilian Rummler, Melanie Krug, Sigrid Müller-Deubert, Martin Lüdemann, Franz Jakob, Bettina M. Willie, Franziska Jundt

**Affiliations:** ^1^Department of Internal Medicine II and Comprehensive Cancer Center Mainfranken, University Hospital of Würzburg, Oberdürrbacher Straße 6, 97080 Würzburg, Germany; ^2^Orthopedic Center for Musculoskeletal Research, University of Würzburg, Friedrich-Bergius-Ring 15, 97076 Würzburg, Germany; ^3^Research Centre, Shriners Hospital for Children-Canada, Department of Pediatric Surgery, McGill University, 1003 Decarie Blvd, Montreal, Canada H4A 0A9

## Abstract

Skeletal development and remodeling of adult bone are critically controlled by activated NOTCH signaling in genetically modified mice. It is yet unclear whether NOTCH signaling is activated by mechanical strain sensed by bone cells. We found that expression of specific NOTCH target genes is induced after *in vivo* tibial mechanical loading in wild-type mice. We further applied mechanical strain through cyclic stretching in human bone marrow-derived mesenchymal stromal cells (BMSCs) *in vitro* by using a bioreactor system and detected upregulation of NOTCH target gene expression. Inhibition of the NOTCH pathway in primary BMSCs as well as telomerase-immortalized human BMSCs (hMSC-TERT) through the gamma-secretase inhibitor GSI XII blocked mechanotransduction and modulated actin cytoskeleton organization. Short-hairpin RNA gene silencing identified NOTCH2 as the key receptor mediating NOTCH effects on hMSC-TERT cells. Our data indicate a functional link between NOTCH activation and mechanotransduction in human BMSCs. We suggest that NOTCH signaling is an important contributor to molecular mechanisms that mediate the bone formation response to mechanical strain.

## 1. Introduction

The NOTCH signaling pathway is evolutionarily highly conserved and regulates cell growth, cell death, and differentiation programs via cell-cell communication [1]. NOTCH receptors (NOTCH1-4) on receiving cells are activated through ligands (JAGGED (JAG1, JAG2) and DELTA-like (DLL1, DLL3, and DLL4)) binding on neighboring cells [1]. After a proteolytic cleavage cascade, the intracellular part of the receptor (NIC) is cleaved involving a *γ*-secretase enzyme activity, which can be blocked by *γ*-secretase inhibitors (GSI) [1]. NIC translocates to the nucleus, binds to recombination signal-binding protein for immunoglobulin kappa J region (RBPjk) and mastermind-like (MAML) proteins in a complex, and controls transcription of canonical NOTCH target genes: the hairy and enhancer of split *HES1* and HES-related with YRPW motif *HEY1*-, *HEY2*-, and HEY-like (*HEYL*) gene families [1].

Conflicting data of studies with global *NOTCH* deletion in mice mainly reflect the apparent cell- and stage-specific function of NOTCH during skeletal development [1]. In young mice, NOTCH signaling maintains the pool of bone marrow-derived mesenchymal stromal cells (BMSCs), the skeletal precursors [2]. In contrast, deletion of members of the *NOTCH* pathway stimulates osteogenic differentiation and trabecular bone formation early on [2], but with aging, the BMSC pool in these knockout mice is diminished, resulting in an osteopenic phenotype. Osteopenia is exacerbated by an overproduction of the osteoclast-stimulating receptor activator of NF-kappaB ligand (RANKL) in mature osteoblasts [2]. One important downstream effector mechanism in this context may be the inhibitory effects of NOTCH target genes *HES1* and *HEY1* on the osteogenic commitment of skeletal precursors, which suppress the transcriptional activity of the core osteogenic transcription factor RUNX2 and the expression of downstream osteogenic marker genes [1]. Conditional overexpression of *NOTCH* in the osteoblastic lineage at various differentiation stages confirms NOTCH's role in maintaining the early differentiation stage of BMSCs. However, conflicting roles of NOTCH signaling in osteocyte development and function were reported: (1) Overexpression of *NOTCH* in mature osteocytes increases bone formation due to an induction of osteoprotegerin (OPG) production and a diminished secretion of the WNT inhibitors sclerostin (SOST) and dickkopf 1 (DKK1). This results in enhanced osteogenic canonical WNT signaling, which is coincident with suppressed bone resorption [3, 4]. (2) Data *in vitro* show that during the transition phase from osteoblasts to osteocytes, a crosstalk between NOTCH and canonical WNT signaling is observed leading to WNT signaling inhibition [5]. Vice versa, osteocyte-specific overexpression of *β*-catenin and subsequent activation of WNT signaling result in increased NOTCH signaling in the bone microenvironment of mice [6].

Active NOTCH further controls bone remodeling processes in mice. Cell-specific activation of NOTCH signaling has anabolic actions on mature bones, promotes bone healing, and prevents tamoxifen-induced bone loss [7]. The transient use of GSI in a murine model of skeletal fracture repairs accelerated bone and cartilage formation via promotion of callus formation and BMSC differentiation, which is coincident with a slight reduction in BMSC numbers [8].

So far, it is unknown whether NOTCH signaling is involved in mechanotransduction in human BMSCs as precursors of human osteoblasts and osteocytes, which may be relevant during fracture healing and bone regeneration. Mechanotransduction as a response to cyclic stretching or fluid flow determines cell fate decision, lineage commitment, and differentiation of mesenchymal precursors. Mechanical loads applied to the bone are converted into a local mechanical signal (widely thought to be strain) that is sensed by bone cells resulting in biochemical cues by use of mechanoreceptors, such as cell membrane-associated proteins, e.g., integrins or calcium channels. As a result, mechanoresponsive signaling cascades such as the mitogen activated protein kinase (MAPK) pathway, which translate mechanical signals into the activation of transcription factors such as AP-1 or SP1, are activated. Both factors bind to responsive elements in the nuclei of effector cells [9–12].

There are hints from the literature that NOTCH signaling might be mechanosensitive and might also influence mechanosensitivity and downstream mechanoresponse. It was shown in a zebrafish model that NOTCH receptor and target gene expression is activated after the application of hemodynamic shear stress during cardiomyocyte trabeculation [13]. With a tension gauge tether assay and by immobilizing the NOTCH ligands JAG1 or DLL1 to a surface, it has been demonstrated that NOTCH signaling is activated by force application in yeast and in *NOTCH1* overexpressing CHO-K1 cells, respectively [14]. Angiogenesis and osteogenesis are defective when blood flow is impaired in vessels of murine long bones, which is coincident with downregulated *NOTCH* signaling in endothelial cells. Artery formation could be rescued by the overexpression of the active *NOTCH1* intracellular domain, and it has been shown that *NOTCH* signaling controls the expression of fluid flow-responsive genes in endothelial cells and modulates the formation of fluid flow-sensing primary cilia [15–18].

In this study, we detect *NOTCH* activation in bone cells after *in vivo* tibial mechanical loading in mice and after cyclic stretching of human BMSCs *in vitro* by use of a small-scale cell culture/bioreactor system.

## 2. Materials and Methods

### 2.1. *In Vivo* Mechanical Loading

RNA was received from wild-type littermate control mice, used in the recently published study by Pflanz et al. [19]. Briefly, the left tibiae of six 10-week-old female C57BL/6 mice underwent a single bout of *in vivo* cyclic compressive loading (216 cycles at 4 Hz, peak strains at a tibial midshaft of +900 *με*) while under anesthesia. Mice were sacrificed at 1 or 24 h after the single loading session, and the right and left tibiae were extracted. Bone marrow was flushed, and RNA was isolated from osteocyte-enriched hard tissue of both limbs using the TRIzol® reagent (Thermo Fisher Scientific, Darmstadt, Germany) followed by purification with an RNeasy kit (QIAGEN GmbH, Hilden, Germany) as described previously [19]. Total RNA was used for first-strand cDNA synthesis with the iScript™ cDNA Synthesis Kit (Bio-Rad Laboratories, Hercules, USA) according to the manufacturer's instructions. Real-time quantitative PCR (qPCR) was performed with the 2x SYBR Green qPCR Master Mix (Bimake, Houston, USA) on a QuantStudio 7 (Thermo Fisher Scientific, Darmstadt, Germany) system. Each reaction was performed in technical duplicate, Ct values were averaged, and relative gene expression was calculated as previously described. Right tibiae served as internal nonloaded controls and were used for normalization. *GAPDH* served as a housekeeping gene. As was previously reported [19], the animal experiments were carried out according to the policies and procedures approved by the local legal research animal welfare representative (LaGeSo, Berlin, G0021/11).

### 2.2. Cell Culture

Primary human BMSCs were obtained from the femoral head of 12 different donors (5 males, 7 females, mean age 63.5 ± 12.6) undergoing elective hip arthroplasty. Material was collected with informed consent from all patients, and the procedure was approved by the local Ethics Committee of the University of Würzburg (06/30/2010). In brief, bone marrow was washed with Dulbecco's modified Eagle's medium (DMEM/F12) (Thermo Fisher Scientific, Darmstadt, Germany) supplemented with 10% fetal calf serum (Bio&Sell GmbH, Feucht, Germany) [20], 100 U/ml penicillin, 0.1 mg/ml streptomycin, and 50 *μ*g/ml ascorbate (Sigma-Aldrich GmbH, Munich, Germany) and centrifuged at 1200 rpm for 5 min. Pellet was washed four times with complete medium, and resulting supernatants containing released cells were collected. Cells were pelleted and cultured at a density of 1 × 10^9^ cells per 175 cm^2^ culture flask. After 2 days, nonattached cells were washed away and adherent ones were cultivated until confluence [21]. HMSC-TERT cells were established from a 33-year-old male donor by the group of Kassem (Odense, Denmark). An AP-1 response element was cloned into the pGL4.14 luciferase reporter vector (Promega, Mannheim, Germany), and the stable hMSC-TERT-AP-1 cell line was generated by electroporation [22]. AP-1 response elements have been shown to be activated by mechanical stimuli. Therefore, the hMSC-TERT-AP-1 clone was used as a tool to perform mechanistic studies. Cells comprising the empty pGL4.14 vector served as controls. HMSC-TERT cells show high proliferation rates, while maintaining their mesenchymal differentiation capacity *in vitro* and *in vivo* [23, 24]. HMSC-TERT-AP-1 cells were cultured in Eagle's MEM supplemented with 10% FCS and 50 *μ*g/ml hygromycin. 293T cells (DSMZ, Braunschweig, Germany) were cultured in Dulbecco's modified Eagle's medium (Thermo Fisher Scientific, Darmstadt, Germany) supplemented with 10% FCS, 100 IU/ml penicillin, and 100 *μ*g/ml streptomycin. All cells were cultivated at 37°C in a humidified 95% air and 5% CO_2_ atmosphere.

### 2.3. Lentiviral Transfer of shRNAs for Knockdown of *NOTCH1* and *NOTCH2* in hMSC-TERT Cells

Lentiviral particles containing short-hairpin RNAs (shRNAs) (designed with the RNAi consortium, TRC (sh*N1*_1 and sh*N1*_2), or obtained from the literature (sh*N2*) [25] for knockdown of *NOTCH1* and *NOTCH2*) were generated by co-transfection of the pLKO (sh*N1*_1 and sh*N1_*2) or the pGIPZ (sh*2*) vector and the packaging plasmids psPAX.2 and pMD2.G into 293T cells. Plasmids were obtained from Addgene (Teddington, UK) and Dharmacon (pGIPZ, Lafayette, USA). In brief, plasmids were mixed with Opti-MEM (Thermo Fisher Scientific, Darmstadt, Germany) and the transfection reagent polyethylenimine (Sigma-Aldrich GmbH, Munich, Germany) and added to 293T cells with 2% FCS-supplemented media overnight. Complete media replaced the transfection mix the next day. Supernatants with virus particles were harvested over two days, filtered, and stored at −80°C. 6 × 10^5^ hMSC-TERT-AP-1 cells were transduced by adding virus supernatants, complete media, and hexadimethrine bromide (Sigma-Aldrich GmbH, Munich, Germany), and 24 h later, complete media replaced the infection mix. Selection with puromycin (Thermo Fisher Scientific, Darmstadt, Germany) started after 24 h and lasted for 3 days. Lentiviral particles with empty vectors or vector expressing nontargeting shRNA were used as controls. Knockdown in transduced cells was confirmed by qPCR for *NOTCH1* and *NOTCH2*. shRNA target sequences were as follows: GAACGAGCATAGTCCAAAAA (sh*N1*_1), GTAGTTGTTCGTTGGTTATA (sh*N1*_2), GCCCAATGTCTCTTGTGACATA (sh*N2*), and TCTCGCTTGGGCGAGAGTAAG (non-targeting control).

### 2.4. Immunoblotting

Whole protein lysates were prepared as previously described [26]. Briefly, 100 *μ*l lysis buffer (50 mM Tris pH 7.5, 150 nN NaCl, 1% Triton, 0.5% NP40, and 0.2% SDS) containing protease inhibitors (Protease Inhibitor Cocktail, Sigma-Aldrich GmbH, Munich, Germany) was used, and protein concentrations were determined using the Roti®-Quant reagent (Carl Roth GmbH, Karlsruhe, Germany). 30 *μ*g protein was mixed with 5 *μ*l loading buffer (375 mM Tris-HCl pH 6.8, 6% SDS, 4.8% glycerol, 9% 2-mercaptoethanol, and 0.03% bromophenol blue) and denatured at 95°C for 5 min. Proteins were separated on 10% polyacrylamide (SDS-PAGE) gels in 0.3% Tris, 0.144% glycin, and 0.5% SDS buffer and transferred to Whatman™ Protran™ Nitrocellulose Blotting Membranes (Thermo Fisher Scientific, Darmstadt, Germany) at 1.5 mA/cm^2^ of membrane for 2 h. Membranes were blocked with 5% SDS in TBS-T buffer (50 mM Tris, 150 mM NaCl, and 0.1% Tween-20) and incubated overnight at 4°C with the following primary antibodies against human epitopes: rabbit anti-NOTCH1 (1 : 1000, Cell Signaling Technology, Frankfurt am Main, Germany), rabbit anti-NOTCH2 (1 : 1000, Cell Signaling Technology, Frankfurt am Main, Germany), mouse anti-HES1 (1 : 2500, Santa Cruz Biotechnology, Heidelberg, Germany), and mouse anti-vinculin (1 : 10000, Sigma-Aldrich GmbH, Munich, Germany). Membranes were washed 3 times for 5 min with TBS-T and incubated for 1 h at room temperature with horseradish peroxidase-conjugated anti-rabbit and anti-mouse IgG (all from Cell Signaling Technology, Frankfurt am Main, Germany) secondary antibodies. After another washing 3 times for 5 min with TBS-T, proteins were visualized using the SuperSignal™ West Dura Extended Duration Substrate (Thermo Fisher Scientific, Darmstadt, Germany) and detection was performed on a ChemiDoc MP Imaging System (Bio-Rad Laboratories, Hercules, USA) using the Image Lab Software (version 5.2.1, Bio-Rad Laboratories, Hercules, USA). All bands were densitometrically analyzed with ImageJ [27].

### 2.5. Cell Viability and Apoptosis Assays

To determine the effects of the NOTCH inhibitor GSI XII (Merck, Darmstadt, Germany) on viability and apoptosis, BMSCs and hMSC-TERT cells were seeded on a 96-well plate with a density of 1000 cells/well. Cells were treated with 2 or 4 *μ*M GSI XII or the solvent DMSO (0 *μ*M) for 24 and 48 h. Viability and apoptosis rates were assessed using the CellTiter-Glo Luminescent Cell Viability Assay and the Caspase-Glo 3/7 Assay, respectively (both from Promega GmbH, Mannheim, Germany), according to the manufacturer's instructions. Luminescence was measured with an Orion II Luminometer (Berthold Detection Systems, Pforzheim, Germany). Data are expressed as mean from triplicates of three independent experiments (hMSC-TERT) or five independent donors (BMSC).

### 2.6. Cyclic Stretching of hMSC-TERT-AP-1 Cells and Luciferase Assay

3 × 10^4^ cells per well were seeded on 24-well polyurethane (PU) plates, and tight cell attachment was observed few hours later, as shown before [22]. After 24 h, cells were treated with the solvent DMSO (indicated as 0 *μ*M) or 2 *μ*M and 4 *μ*M GSI XII, and after incubation for 24 h, PU dishes were placed in a bioreactor [22]. Cyclic stretching was applied twice for 30 min (1 Hz and 1% extension), with a 60 min pause in between. After 24 h, cells were lysed in 150 *μ*l Reporter Lysis Buffer (Promega GmbH, Mannheim, Germany) and 20 *μ*l of extracts was used for the measurement of luciferase activity with the reporter gene assay (Promega GmbH, Mannheim, Germany) in an Orion II Luminometer (Berthold Detection Systems, Pforzheim, Germany). Protein content was determined with the Roti-Quant Protein Assay (Carl Roth GmbH, Karlsruhe, Germany) and used for normalization of luminescence units. Four technical replicates were obtained from four independent wells.

### 2.7. Cyclic Stretching of BMSCs

BMSCs were obtained from different donors as indicated, and 5 × 10^5^ cells per well were seeded on 4-well PU plates, allowed to attach, and cultured for one week. Cells were treated with 2 and 4 *μ*M GSI XII or the solvent DMSO (0 *μ*M) for 24 h. PU dishes were placed in a bioreactor, and cyclic stretching was applied as previously described [10, 12]. 15 min and 4 h later, cells were harvested and total RNA was isolated by using the NucleoSpin RNA II kit (Macherey-Nagel, Düren, Germany) according to the manufacturer's instructions.

### 2.8. Reverse Transcription and qPCR Analysis

For mRNA reverse transcription, one microgram of total RNA was used for first-strand cDNA synthesis with MMLV reverse transcriptase (Promega GmbH, Mannheim, Germany) in 25 *μ*l total volume as previously described [28]. cDNA was diluted to 1 : 10, and 2 *μ*l was used for real-time qPCR with the GoTaq qPCR Master Mix (Promega GmbH, Mannheim, Germany) in a 20 *μ*l total volume. Sequence-specific primers (5 pmol per reaction) were designed using the Universal ProbeLibrary System Assay Design (Roche, Mannheim, Germany). Primers were obtained from QIAGEN GmbH (Hilden, Germany) or from published studies as indicated (see Table 1 for primer sequences and PCR conditions). Each reaction was performed in technical triplicate, and Ct values were averaged. Relative gene expression was calculated with the efficiency-corrected Ct model [29] with *RPS27A* as the housekeeping gene [30].

### 2.9. Actin Cytoskeleton Staining with Phalloidin

2000 BMSCs per cm^2^ were seeded on 12-well plates and grown overnight. Cells were treated with 2 or 4 *μ*M GSI XII or the solvent DMSO for 24 or 48 h, respectively, washed with PBS, and fixed with 4% paraformaldehyde for 10 min. After three additional PBS washing steps, cells were incubated with 0.5% Triton X-100 in PBS for 5 min and washed again three times with PBS. Afterwards, cells were added with 3% nonfat dry milk in PBS as blocking solution, incubated for 30 min, and rinsed with PBS. Cells were incubated with phalloidin staining solution (5 units of phalloidin CF488A/ml 1% BSA-PBS, Linaris GmbH, Dossenheim, Germany) for 20 min in the dark and washed three times with PBS. Cells were mounted with VECTASHIELD mounting medium with DAPI, stored at 4°C, and protected from light. Phalloidin staining was analyzed by fluorescence microscopy with a Leica DMi8 microscope (Leica Microsystems, Wetzlar, Germany).

### 2.10. Statistical Analyses

Statistical analyses were performed using two-tailed unpaired or paired *t*-test, and *P* values less than 0.05 were considered significant. All values were obtained from at least three technical replicates, except for qPCR analysis of murine mRNA which was performed in duplicate and expressed as mean ± SD. Asterisks indicate significant differences against control samples used for normalization (dashed line), and hash sign indicates significant differences between samples (compared samples are annotated with connecting lines). Further details of the number of independent experiments, BMSC donors used, and selection of the normalization method are given in the figure legends.

## 3. Results

### 3.1. NOTCH Target Gene Activation in Osteocyte-Enriched Bone after *In Vivo* Tibial Mechanical Loading

**Figure 1:**
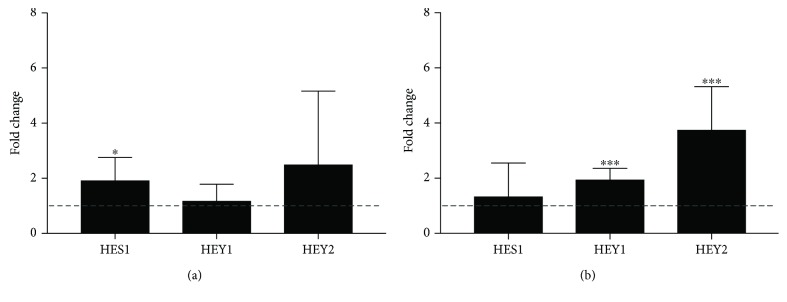
Gene expression of *HES1*, *HEY1*, and *HEY2* 1 and 24 h after a single loading session in left-loaded and right-nonloaded osteocyte-enriched tibiae of 10-week-old female C57BL/6 mice. Fold changes in gene expression of loaded limbs (*n* = 6) normalized to control limbs are shown (a) 1 h and (b) 24 h after loading. qPCR data were obtained from technical duplicates. Results are shown as mean ± SD; fold change was calculated with the ΔΔCt method and normalized to basal expression (nonloaded right limb, dashed line). *GAPDH* served as the housekeeping gene. Two-tailed paired *t*-test was used for statistical analysis (^∗^*P* < 0.05 and ^∗∗∗^*P* < 0.001).

To evaluate NOTCH activation after tibial mechanical loading in mice, we performed qPCR analysis of NOTCH target genes in osteocyte-enriched bone from left-loaded and right-nonloaded limbs [19]. 1 and 24 h after a single loading session, we detected up to 6-fold increases in mRNA expression of the NOTCH targets *HES1*, *HEY1*, and *HEY2* dependent on the time point (Figures 1(a) and 1(b)).

### 3.2. NOTCH Signaling Is Activated in BMSCs after Cyclic Stretching

**Figure 2:**
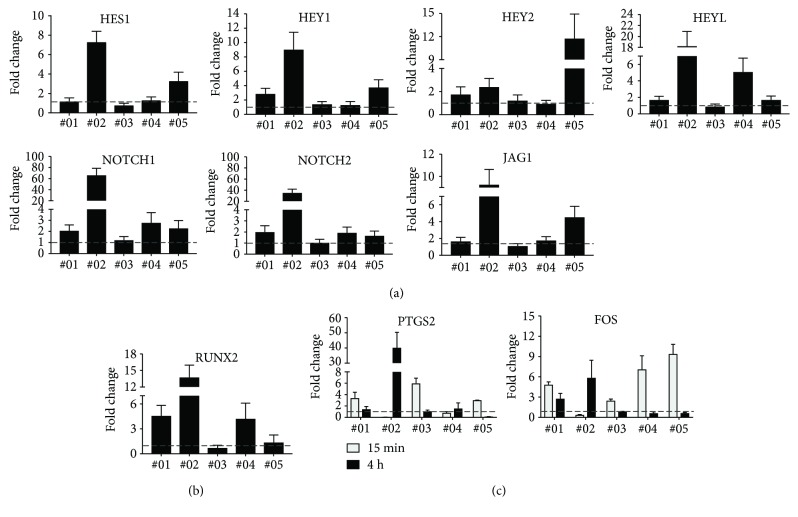
NOTCH target gene expression after cyclic stretching of BMSCs derived from five donors (donor #01-donor #05). (a) Relative mRNA expression of the NOTCH target genes *HES1*, *HEY1*, *HEY2*, and *HEYL*; the *NOTCH1* and *NOTCH2* receptor genes; and the NOTCH ligand gene *JAG1*. (b) Relative mRNA expression of the early osteogenic transcription factor RUNX2. Samples in (a) and (b) were collected 4 h after cyclic stretching. (c) Relative mRNA expression of the mechanoresponsive genes *PTGS2* and *FOS* was analyzed 15 min and 4 h after cyclic stretching. qPCR data were obtained from technical triplicates (a, b) or from technical triplicates derived from three independent qPCR (c). Results are shown as mean ± SD; fold change was calculated with the ΔΔCt method and normalized to basal activity (nonstretched, dashed line). *RPS27A* served as the housekeeping gene.

To clarify if NOTCH signaling is responsive for mechanical strain in osteogenic precursors, BMSCs from five donors were seeded on PU dishes and mechanical strain (1 Hz, 1%) was applied. In donor #02, gene expression of the NOTCH receptors *NOTCH1* and *NOTCH2* was induced over 60-fold and 30-fold, respectively; three donors (#1, #4, and #5) responded with a 2- to 3-fold increase in *NOTCH1* and *NOTCH2* expression (Figure 2(a)). Additionally, expression of the NOTCH target genes *HES1*, *HEY1*, *HEY2*, and *HEYL* was quantified. Donor #02 depicted the highest induction of *HES1* (7-fold), *HEY1* (9-fold), and *HEYL* (18-fold), whereas donor #05 showed the highest increase in *HEY2* expression (12-fold) after cyclic stretching. In two donors, expression of *HEY1* (#1 and #5) and *HEY2* (#1 and #2) was upregulated 2- to 3-fold, and in one donor, *HES1* (#5) and *HEYL* (#4) gene expression was increased 3- to 5-fold. The NOTCH ligand *JAG1* was also increased after mechanical stimulation. Donor #02 depicted a 9-fold increase and donor #05 a 4-fold increase. Analysis of the early osteogenic transcription factor *RUNX2* revealed a more than ten-fold upregulation in donor #02 and a 4-fold induction in donor #01 and donor #04 (Figure 2(b)). Overall, application of cyclic strain to BMSCs induced expression of *NOTCH1*; *NOTCH2*; the NOTCH ligand *JAG1*; the NOTCH target genes *HES1*, *HEY1*, *HEY2*, and *HEYL*; and the early osteogenic transcription factor *RUNX2* with high donor variability.

To confirm that BMSCs are mechanosensitive and respond to cyclic stretching expression of the mechanoresponsive genes, *PTGS2* and *FOS* [9, 33] were analyzed in the same donors 15 min and 4 h after mechanical loading (Figure 2(c)). While *PTGS2* was induced early after 15 min in donor #03 and donor #05, donor #02 responded later at 4 h. *FOS* gene expression was induced after 15 min in donors #01, #03, #04, and #05, while donor #02 showed the highest induction 4 h after cyclic stretching. qPCR revealed a donor- and time-dependent induction of *PTGS2* and *FOS*, while all donors responded to cyclic strain and were mechanoresponsive.

### 3.3. NOTCH Inhibition Does Not Affect Cell Viability and Apoptosis of BMSCs

**Figure 3:**
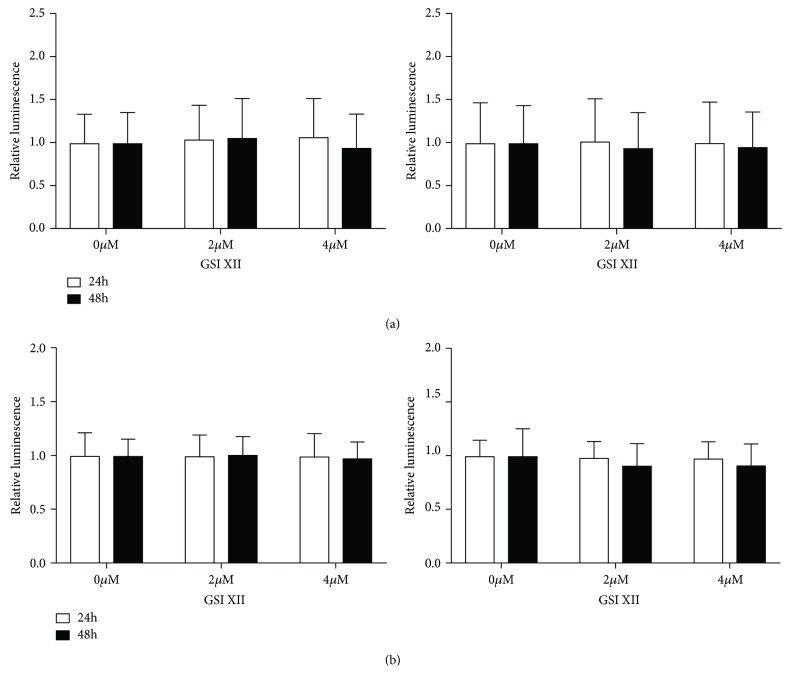
Effect of GSI XII on viability and apoptosis of hMSC-TERT cells and BMSCs derived from five donors (donor #06-donor #10). hMSC-TERT cells (a) and BMSCs (b) were treated with 2 and 4 *μ*M of GSI XII or the solvent DMSO (0 *μ*M), and viability and apoptosis assays were performed 24 and 48 h later. Relative luminescence is given. Data are expressed as mean of three independent experiments ± SD (a) or mean of five different donors ± SD (b) and normalized to untreated control. Each measurement was performed in technical triplicate. Two-tailed unpaired *t*-test was used for statistical analysis.

In a next step, we blocked NOTCH signaling through the gamma-secretase inhibitor GSI XII in BMSCs and hMSC-TERT cells. BMSCs and hMSC-TERT cells were treated with 2 and 4 *μ*M of GSI XII or the solvent DMSO (0 *μ*M). GSI XII doses were used for NOTCH inhibition as described previously [34]. Neither hMSC-TERT cells (Figure 3(a)) nor primary BMSCs (Figure 3(b)) showed a decrease in cell viability or an increase in apoptosis after 24 or 48 h, respectively. Therefore, we determined GSI XII doses of 2 and 4 *μ*M as suitable for NOTCH inhibition without cell toxic side effects.

### 3.4. NOTCH Inhibition Impairs Mechanotransduction in BMSCs and hMSC-TERT Cells

**Figure 4:**
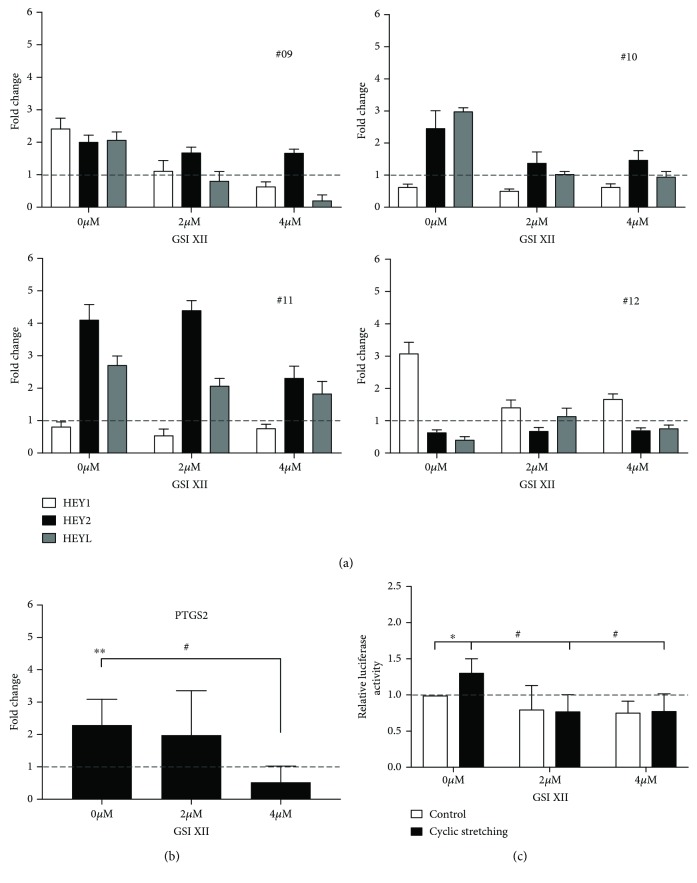
Effect of NOTCH inhibition on mechanotransduction (donor #09-donor #12). (a) Relative mRNA expression of the NOTCH target genes *HEY1*, *HEY2*, and *HEYL* in BMSCs from four representative donors after stretching and GSI inhibition as described in (b). Results are shown as mean of technical triplicates ± SD and normalized to basal activity (DMSO treated, nonstretched, dashed line). Fold change was calculated with the ΔΔCt method, and *RPS27A* served as the housekeeping gene. (b) Relative mRNA expression of the *PTGS2* gene in BMSCs. Cells were treated with 2 *μ*M and 4 *μ*M GSI XII or the solvent DMSO and received cyclic stretching. Samples were collected 4 h later. Results are shown as mean of four donors (#09-12)±SD and normalized to basal activity (DMSO or GSI XII treated, nonstretched, dashed line). Fold change was calculated with the ΔΔCt method, and *RPS27A* served as the housekeeping gene. Two-tailed unpaired *t*-test was used for statistical analysis (^∗∗^*P* < 0.01: stretched compared to nonstretched, ^#^*P* < 0.01: DMSO control compared to 4 *μ*M GSI XII). (c) hMSC-TERT-AP-1 cells were treated with 2 *μ*M and 4 *μ*M GSI XII or DMSO as solvent and received cyclic stretching. Luciferase activity was determined and normalized to protein content. Data are expressed as mean of four independent experiments ± SD and normalized to nonstretched, untreated controls (first column). Each measurement was performed in technical quadruplicate (^∗^*P* < 0.05: stretched compared to nonstretched, #*P* < 0.05: DMSO control compared to 2 *μ*M or 4 *μ*M GSI XII).

To evaluate if NOTCH inhibition has an impact on mechanotransduction in primary BMSCs, cells were seeded on PU dishes and pretreated with 2 and 4 *μ*M GSI XII or the solvent DMSO for 24 h and mechanical strain was applied. Expression of the NOTCH target genes *HEY1*, *HEY2*, and *HEYL* was analyzed by qPCR.  Figure 4(a) shows results of four representative donors. In donor #09, mechanodriven induction of *HEY1* and *HEYL* (0 *μ*M GSI XII) was blunted by GSI XII dose-dependently while expression of *HEY2* was slightly influenced (Figure 4(a)). In donors #10 and #11, expression of *HEY2* and *HEYL* was induced after cyclic stretching (0 *μ*M GSI XII) and inhibited through GSI XII whereas *HEY1* expression (#12, #13) was not altered. In donor #12, the NOTCH target *HEY1* was upregulated (0 *μ*M GSI XII) 3-fold after mechanical stimulation and no longer responded after NOTCH inhibition.

To analyze if the induction of a known and established mechanoresponsive gene in primary BMSCs could be reversed by NOTCH inhibition, expression of *PTGS2* was analyzed by qPCR after pretreatment with GSI XII and application of cyclic strain. Mechanoresponse of *PTGS2* was abolished significantly by GSI XII in a dose-dependent manner (Figure 4(b)).

By using hMSC-TERT, comprising a mechanoresponsive AP-1-driven luciferase reporter (hMSC-TERT-AP-1), we analyzed if NOTCH inhibition had an impact on the AP-1-mediated mechanoresponse. As reported before [22], AP-1-controlled luciferase activity was induced significantly by cyclic strain (Figure 4(c)). Induction of luciferase activity was abolished by 2 and 4 *μ*M GSI XII (black bars). No effect of GSI XII could be observed on hMSC-TERT-AP-1 cells that were grown without mechanical loading (white bars).

### 3.5. Phalloidin Staining Reveals a Cytoskeletal Modulation upon NOTCH Inhibition

**Figure 5:**
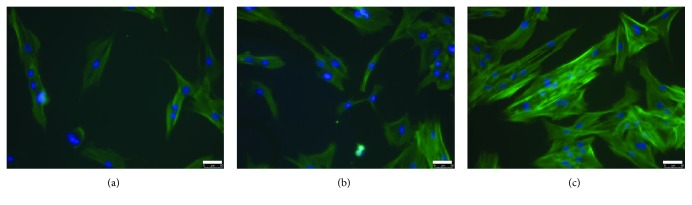
Phalloidin staining (green) of BMSCs after treatment with the NOTCH inhibitor GSI XII. Cells were incubated with 2 *μ*M (b) and 4 *μ*M (c) GSI XII for 24 h; DMSO-treated cells served as a control (a). Representative images of one donor of three independent experiments are shown. DAPI (blue) was used for nuclear staining. The bar represents 50 *μ*m.

We next investigated if cytoskeletal organization and actin remodeling, which mediate mechanotransduction in cells, are affected by NOTCH signaling at basal conditions (nonstretched). In untreated control BMSCs, phalloidin staining revealed a diffuse cytoskeleton organization (Figure 5(a)). After treatment of BMSCs with 2 and 4 *μ*M of GSI XII (Figures 5(b) and 5(c)) for 24 h, the cytoskeleton depicted more structured actin fibers compared to untreated cells. In addition, GSI XII treatment resulted in a brighter phalloidin staining indicating a higher actin content or a denser and stiff arrangement of actin fibers.

### 3.6. The NOTCH2 Receptor Mediates NOTCH Signaling in hMSC-TERT Cells

**Figure 6:**
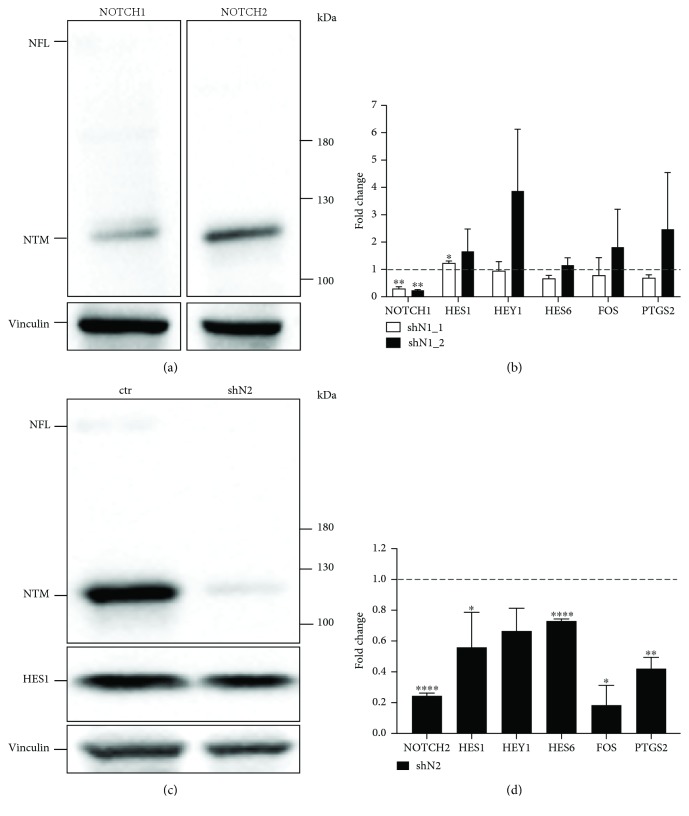
Knockdown of *NOTCH1* and *NOTCH2* genes in hMSC-TERT-AP-1 cells. (a) Immunoblotting of whole cell lysates of hMSC-TERT-AP-1 cells with antibodies against NOTCH1 and NOTCH2. Full-length NOTCH (NFL), transmembrane and intracellular part of NOTCH (NTM), and housekeeping protein vinculin. (b) hMSC-TERT-AP-1 cells were stably transduced with lentiviral vectors expressing shRNAs against *NOTCH1* (sh*N1*_1 and sh*N1*_2). Relative mRNA expression of *NOTCH1*, *HES1*, *HEY1*, *HES6*, *FOS*, and *PTGS2* is shown. Results are given as mean of two independent experiments ± SD and normalized to an empty vector (dashed line). (c) Immunoblotting of whole cell lysates with antibodies against NOTCH2 after transfer of shN2 and nontargeting shRNA (ctr). (d) Relative mRNA expression of *NOTCH2*, *HES1*, *HEY1*, *HES6*, *FOS*, and *PTGS2* after transfer of sh*N2* compared to nontargeting shRNA (dashed line). Two-tailed unpaired *t*-test was used for statistical analysis (^∗^*P* < 0.05, ^∗∗^*P* < 0.01, and ^∗∗∗∗^*P* < 0.0001). qPCR data were obtained from technical triplicates.

Next, we investigated which NOTCH receptor (NOTCH1 or NOTCH2) mainly mediates signaling in BMSCs. We found that the transmembrane part of the NOTCH2 receptor (NTM) is expressed higher (up to 2-fold) than that of the NTM of NOTCH1 in hMSC-TERT-AP-1 cells (Figure 6(a)). Figure 6(a) shows a representative blot where densitometrical analysis of NTM normalized to the expression of the housekeeping protein vinculin was performed. To study specific NOTCH effects on nonstretched hMSC-TERT-AP-1 cells, we performed lentivirally mediated shRNA knockdown studies of NOTCH1 and NOTCH2 receptors (Figures 6(b) and 6(d)). Efficient mRNA knockdown of *NOTCH1* (sh*N1*_1, sh*N1*_2, Figure 6(b)) and *NOTCH2* (sh*N2*, Figure 6(d)) was confirmed by qPCR analysis. In addition, immunoblotting revealed reduction of NOTCH2 (sh*N2*: 0.08-fold compared to ctr; normalized to vinculin) and *HES1* (sh*N2*: 0.76-fold compared to ctr; normalized to vinculin) protein levels after shRNA transfer (Figure 6(c)). We further evaluated the mRNA expression of NOTCH target (*HES1*, *HEY1*, and *HES6*) and mechanoresponsive (*FOS*, *PTGS2*) genes after specific *NOTCH1* and *NOTCH2* knockdown compared to control plasmid in hMSC-TERT-AP-1 cells (Figures 6(b) and 6(d)). *NOTCH2* knockdown revealed significant downregulation of the NOTCH target genes *HES1* and *HES6* and the mechanoresponsive genes *FOS* and *PTGS2* (Figure 6(d)). *NOTCH1* knockdown with two different shRNAs (sh*N1*_1 and sh*N1*_2) showed almost no significant effects on target and mechanoresponsive genes (Figure 6(b)).

## 4. Discussion

In this study, we evaluated expression of the NOTCH target genes *HES1*, *HEY1*, and *HEY2* in murine tibial osteocyte-enriched bones after mechanical stimulation. No previous studies have reported NOTCH signaling after *in vivo* mechanical loading as much more attention has been focused on the WNT signaling pathway [35–38]. Our data indicate that NOTCH signaling is activated in bone cells as part of the anabolic mechanoresponse. We further evaluated whether osteoblastic precursors, mechanosensitive BMSCs, are a target of NOTCH activity in the bone. To that end, we used our small-scale cell culture/bioreactor system and applied cyclic stretching to analyze subcellular crosstalk mechanisms in mechanotransduction of human primary BMSCs and immortalized hMSC-TERT cells. In individual BMSC preparations, mRNA expression of NOTCH target genes such as *HES1*, *HEY1*, *HEY2*, and *HEYL* and the *NOTCH1* and *NOTCH2* genes and their ligand *JAG1* was commonly increased after cyclic stretching although high donor variability was observed. To verify if all used BMSC donors responded to mechanical loading, we harvested RNA of BMSCs 15 min and 4 h after cyclic stretching and amplified the known mechanoresponsive genes *PTGS2* [33] and *FOS* [9]. As expected *PTGS2* and *FOS* were upregulated after mechanical loading in all donors, dependent on the time point.

Inhibition of the NOTCH pathway in BMSC donors through GSI XII abolished the upregulation of NOTCH target genes after cyclic stretching. Moreover, upregulation of *PTGS2* after cyclic stretching in those BMSC donors and induction of the mechanosensitive AP-1-driven luciferase reporter in hMSC-TERT cells [22] were blocked by GSI XII in a dose-dependent manner. Our findings indicate that NOTCH signaling controls expression of mechanoresponsive genes in BMSCs. We therefore suggest that activation of the NOTCH pathway is part of the mechanotransduction process in BMSCs. To exclude cytotoxic effects of GSI XII on primary BMSCs or hMSC-TERT cells, we performed cell viability and apoptosis assays. No cell toxic effects of GSI XII were observed.

To clarify if *NOTCH1*, *NOTCH2*, or both receptors mediate the effects on BMSCs, we transduced hMSC-TERT-AP-1 cells with lentiviral knockdown constructs for *NOTCH1* and *NOTCH2*. MRNA expression of *NOTCH* target genes and the mechanoresponsive *FOS* and *PTGS2* genes were significantly downregulated in nonstretched hMSC-TERT cells after knockdown of *NOTCH2*. Our data reveal NOTCH2 as the key receptor, which mediates NOTCH effects on BMSCs.

The recent literature about the role of NOTCH signaling in bone biology favors a two-step model: (1) NOTCH signaling in early skeletal precursors inhibits osteogenic commitment and amplifies the precursor pool and (2) activation in later stages after osteoblast-to-osteocyte transition stimulates bone formation and mineralization via downregulation of the WNT inhibitors SOST and DKK1 and upregulation of the RANKL decoy receptor OPG [7]. Thereby, both precursor-specific and osteocyte-specific NOTCH activation may overcome age-associated osteopenia and prevent post-ovariectomy-induced bone loss. In the setting of fracture healing, NOTCH signaling promotes bone healing following osteotomy in mice [7]. It is well known that callus formation and fracture healing are markedly influenced by mechanical cues [39, 40]. Our *in vitro* data provide a first functional link between NOTCH activation and the mechanoresponse of mechanosensitive skeletal precursors (BMSCs). A recent report described that in an osteoblast cell line, compressive forces enhanced NOTCH target gene expression via TGF*β* signaling [41, 42]. We suggest that BMSCs as precursors of osteoblasts and osteocytes contribute to the mechanoresponse of the early phases of bone regeneration and fracture healing through activation of NOTCH.

During mechanotransduction, extracellular signals are transduced via mechanoresponsive surface proteins from the outer cell membrane to the cytosol and the nucleus [43]. As the cytoskeleton is involved in this process and actin fibers are mediating the effects, we investigated if active NOTCH signaling is needed for proper cytoskeleton or actin organization. Interestingly, the actin cytoskeleton organization, as evidenced by phalloidin staining, was modulated through the NOTCH inhibitor GSI XII in a dose-dependent manner. We suggest that stiffening of actin fibers prevents mechanotransduction in BMSCs and that NOTCH activation is needed for cytoskeleton organization and function. Published data demonstrate a link between NOTCH signaling and cytoskeletal organization and actin remodeling in other cell types. In a zebrafish model, it has been shown that inhibition of NOTCH signaling led to a loss of stress fibers in the endoskeletal disc cells of the pectoral fin [44]. Overexpression of NIC in endothelial cells resulted in generation of stress fibers [45], and in hepatocellular carcinoma cells, application of actin cytoskeletal modulators resulted in activation of NOTCH signaling and induction of epithelial mesenchymal transition [46]. In BMSCs, our data indicate that active NOTCH signaling and the organization of the actin cytoskeleton are linked; however, the mechanism remains elusive.

Here, we present a first indication of how NOTCH signaling contributes to the complex network of transducing mechanosensitivity in BMSCs and we identified *NOTCH2* as the key NOTCH receptor in BMSCs. Our findings provide a functional link between NOTCH activation and mechanotransduction in BMSCs as mediators of the anabolic response in bones.

## 5. Conclusion

NOTCH signaling is activated through mechanical strain in human BMSCs and after *in vivo* tibial mechanical loading in bone cells of wild-type mice. NOTCH controls expression of mechanoresponsive genes and is part of the mechanotransduction process in BMSCs.

## Figures and Tables

**Table 1:** Primer names, sequences, product lengths, annealing temperatures, and GenBank accession numbers are shown.

Gene name	Primer	Sequence 5′-3′	Product length	Annealing temp (°C)	GenBank accession number
Primers designed by the Universal ProbeLibrary System Assay					
HES1	HES1 FOR_h	GGAAATGACAGTGAAGCACCT	78	63.7	NM_005524.2
	HES1 REV_h	CAGCACACTTGGGTCTGTG			
HEY1	HEY1 FOR_h	GGCAGGAGGGAAAGGTTACT	79	53.6	NM_001040708.1
	HEY1 REV_h	CTCAGATAACGCGCAACTTC			
HEY2	HEY2 FOR_h	CCCGCCCTTGTCAGTATC	60	54.5	NM_012259.2
	HEY2 REV_h	TTGTTTGTTCCACTGCTGGT			
HEYL	HEYL FOR_h	GTCCCCACTGCCTTTGAG	88	54.9	NM_014571.3
	HEYL REV_h	ACCGTCATCTGCAAGACCTC			
JAG1	JAG1 FOR_h	GGCAACACCTTCAACCTCA	103	55.5	NM_000214.3
	JAG1 REV_h	GCCTCCACAAGCAACGTATAG			
NOTCH1	NOTCH1 FOR_h	CGGGGCTAACAAAGATATGC	68	54.5	NM_017617.3
	NOTCH1 REV_h	CACCTTGGCGGTCTCGTA			
NOTCH2	NOTCH2 FOR_h	TGGTGGCAGAACTGATCAAC	78	63.4	NM_024408.3
	NOTCH2 REV_h	CTGCCCAGTGAAGAGCAGAT			
RPS27A	RPS27A FOR_h	TCGTGGTGGTGCTAAGAAAA	141	60	NM_001135592
	RPS27A REV_h	TCTCGACGAAGGCGACT			
RUNX2	RUNX2 FOR_h	CTTCACAAATCCTCCCCAAG	147	58	NM_001024630.3
	RUNX2 REV_h	ATGCGCCCTAAATCACTGAG			
HES1	HES1 FOR_m	CGGTCTACACCAGCAACAGT	88	66	NM_008235.2
	HES1 REV_m	CACATGGAGTCCGAAGTGAG			
HEY1	HEY1 FOR_m	CCTTTGAGAAGCAGGGATCT	142	65	NM_010423.2
	HEY1 REV_m	CCCAAACTCCGATAGTCCAT			
Primer sequences obtained from published studies					
HEY2 [31]	HEY2 FOR_m	TCCACCTCTCTTCTGTCTCTTTCG	190	69	NM_013904.1
	HEY2 REV_m	GACTGGAGGCTGCGGATACC			
GAPDH [32]	GAPDH_FOR_m	TGCGATGGGTGTGAACCACGAGAA	130	68.9	NM_008084.3
	GAPDH_REV_m	GAGCCCTTCCACAATGCCAAAGTT			
Primers obtained from QIAGEN					
PTGS2	Hs_PTGS2_1_SG	QIAGEN sequence		59	NM_000963
FOS	Hs_FOS_1_SG	QIAGEN sequence		57	NM_005252.3

## Data Availability

The raw data of the qPCR and viability assays used to support the findings of this study are available from the corresponding author upon request.
